# Regulation of alfalfa growth, water and nitrogen utilization and distribution in arid region of Northwest China by optimizing irrigation method

**DOI:** 10.3389/fpls.2025.1517398

**Published:** 2025-03-12

**Authors:** Hongxiu Ma, Peng Jiang, Xiaojuan Zhang, Ruliang Liu, Quan Sun, Lei Wang

**Affiliations:** ^1^ Breeding Base for State Key Laboratory of Land Degradation and Ecological Restoration in northwestern China; Key Laboratory of Restoration and Reconstruction of Degraded Ecosystems in northwestern China of Ministry of Education, Ningxia University, Yinchuan, Ningxia, China; ^2^ College of Forestry and Prataculture, Ningxia University, Yinchuan, Ningxia, China; ^3^ Institute of Agricultural Resources and Environment, Ningxia Academy of Agro-forestry Science, Yinchuan, Ningxia, China; ^4^ School of Ecology and Environment, Ningxia University, Yinchuan, Ningxia, China

**Keywords:** nitrogen use efficiency, root-shoot ratio, subsurface drip irrigation, water use efficiency, NH3 volatilization

## Abstract

The water and nitrogen use efficiency of alfalfa is very low in the arid region of Northwest China currently. In this field experiments in 2022 and 2023, the effects of traditional flood irrigation (FI-12, 1200 mm; FI-8, 880 mm), sprinkler irrigation (SI-8, 880 mm; SI-5, 520 mm), and subsurface drip irrigation (DI-5, 520 mm; DI-8, 880 mm)) on alfalfa yield, water use efficiency (WUE), and nitrogen use efficiency (NUE) were studied. The results showed that the DI and SI treatments, especially DI-5, increased alfalfa seed yield by increasing the number of inflorescences and pods compared with the FI treatments. The DI and SI treatments, especially DI, reduced water loss during the first two crops in each growing season compared with the FI treatments, improving the WUE. The DI treatments had the lowest root/shoot ratio (R/S), which facilitated the distribution of photosynthetic products to the reproductive organs and inhibited the overgrowth of the root system. The small R/S in the late growth stage of the DI-5 treatment also helped to achieve high WUE. Besides, the DI treatments also had the largest root length density, which promoted the uptake and utilization of water and nitrogen by alfalfa. The DI treatments increased the nitrogen accumulation of plants, and reduced the soil nitrate (NO_3_^−^-N) leaching and NH_3_ volatilization at maturity stage compared with the SI and FI treatments, improving the NUE. In summary, the subsurface drip fertigation, especially DI-5, coordinated the vegetative and reproductive growth, and reduced the water loss, nitrate leaching, and NH_3_ volatilization, improving the seed yield, WUE, and NUE of alfalfa. This study will advance understanding of the mechanism of subsurface drip irrigation regulating alfalfa root growth and water and nitrogen use, and provide a scientific basis for the application of subsurface drip fertigation in arid and semi-arid areas.

## 1 Introduction

Ningxia Yellow River Irrigation Area is one of the most important livestock production bases of China and a major production area of high-quality forage, with an alfalfa planting area of 400,000 hm^2^ (National Bureau of Statistics of China, 2015). However, in the face of the decrease in the water volume of the Yellow River and the increase in non-agricultural water demand, the shortage of agricultural water resources has greatly limited the sustainable development of local agriculture (Pereira et al., 2007).

Alfalfa root, responsible for absorbing water and nutrients, is vital for alfalfa growth and yield (Yu et al., 2006; Sun et al., 2024). Water and nitrogen uptake and utilization have a direct effect on the vertical distribution of alfalfa roots in soil (Bucciarelli et al., 2021; Pan et al., 2023). Gherardi and Rengel (2003) reported that the root system of alfalfa was mainly distributed in the 0-75 cm soil layer, and the roots distributed in the 0-30 cm soil layer accounted for 75%-95% (Josef et al., 2017). Some studies have found that optimizing the irrigation strategy could promote the growth of plant root systems in the deep soil, thereby enhancing the ability to absorb and utilize deep soil water and improving WUE (Thomas et al., 2012; Amir et al., 2013). In addition, proper root distribution can also increase the NUE of crops, and reduce the loss of nitrogen and the negative impacts of nitrogen loss on the environment (Scott et al., 2022).

The traditional fertilization method for farmers in Northwest China to grow alfalfa is to apply nitrogen, phosphorus, and potassium fertilizers at the time of sowing, and nitrogen fertilizer through the irrigation system after the first two crops. In sandy soils with large pores and good aeration, nitrogen fertilizers are rapidly converted to nitrate nitrogen (NO_3_^−^-N) through nitrification, and easily leach into the deep soil, especially under over irrigation conditions, polluting groundwater and increasing greenhouse gas emissions (Gheysari et al., 2009; Mushtaq et al., 2015; Li et al., 2022; Scott et al., 2022). Traditional agricultural production methods are difficult to achieve precise irrigation and fertilization, which reduces the efficiency of water and fertilizer use.

Flood irrigation, characterized by large water consumption, poor uniformity, difficult water control, and large water loss by evaporation, is still used as the main irrigation method in this area. This irrigation method can easily lead to soil compaction and secondary salinization, and reduce forage yield and WUE (Mitchell and Van Genuchten, 1993; Hu et al., 2016). Subsurface drip irrigation is a kind of micro-irrigation. Subsurface drip fertigation can directly supply water and nutrients to crop roots, and significantly reduce the water loss by evaporation and fertilizer loss. Therefore, subsurface drip irrigation is conducive to increasing crop yield and reducing farm costs (Dukes and Scholberg, 2005; Du et al., 2017). Previous studies have shown that under the premise of equal yields, subsurface drip irrigation saved 50%-60% and 20%-30% of water compared with furrow irrigation and surface drip irrigation, respectively (Yang et al., 2000; Han et al., 2019). Different from sprinkler irrigation, the subsurface drip irrigation system is hidden deep in the soil so as not to affect the mechanical harvesting. Besides, there is no need to stop irrigation and fertilization before and after each mowing due to the fear of mildew, which prolongs the growth period and helps increase yield. Therefore, the large-scale application of subsurface drip irrigation is of great practical significance for the efficient utilization of water and fertilizer resources and the improvement of alfalfa yields and farmland environment in arid and semi-arid areas of China.

To explore whether the root and shoot growth of alfalfa can be adjusted through the subsurface drip irrigation, so as to achieve efficient water and nitrogen utilization and high yields, a two-year field experiment was conducted. This study hypothesized that (1) subsurface drip irrigation might directly supply water and nitrogen to the root zone, inhibit the over growth of alfalfa root and shoot, and increase seed yield, compared with the traditional flood and sprinkler irrigation. (2) Subsurface drip fertigation might ensure the water and fertilizer nutrient supply for the root zone during the key growth period of alfalfa, reduce the soil nitrate nitrogen leaching, and increase the uptake and utilization of water and nitrogen by alfalfa. The specific objectives were to clarify the effects of different irrigation methods on alfalfa yield (seed yield, dry matter yield, and harvest index), water use (water consumption, WUE, and soil moisture content (SMC)), root distribution, and nitrogen use (soil NO_3_^−^-N content, NH_3_ volatilization, crop nitrogen accumulation, and nitrogen use efficiency). This study will provide a scientific basis for the application of subsurface drip irrigation technology in arid and semi-arid areas, promote the efficient use of water and fertilizer resources, and alleviate the negative impacts of agricultural production on the environment.

## 2 Materials and methods

### 2.1 Experimental site

**Figure 1:**
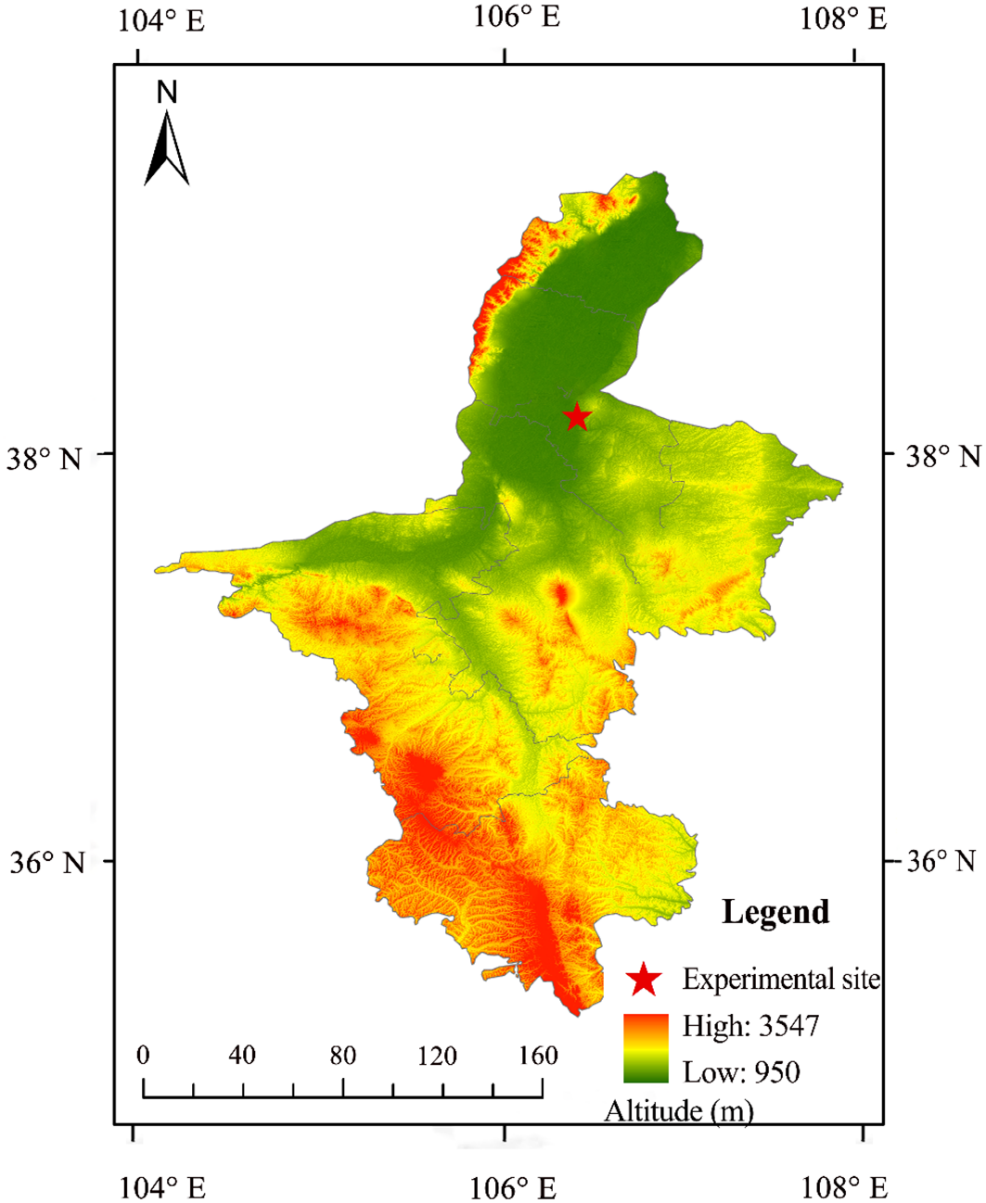
Location of the experimental site. The maps are drawn using Arcgis software v.10.2 (http://www.esri.com/).

**Figure 2:**
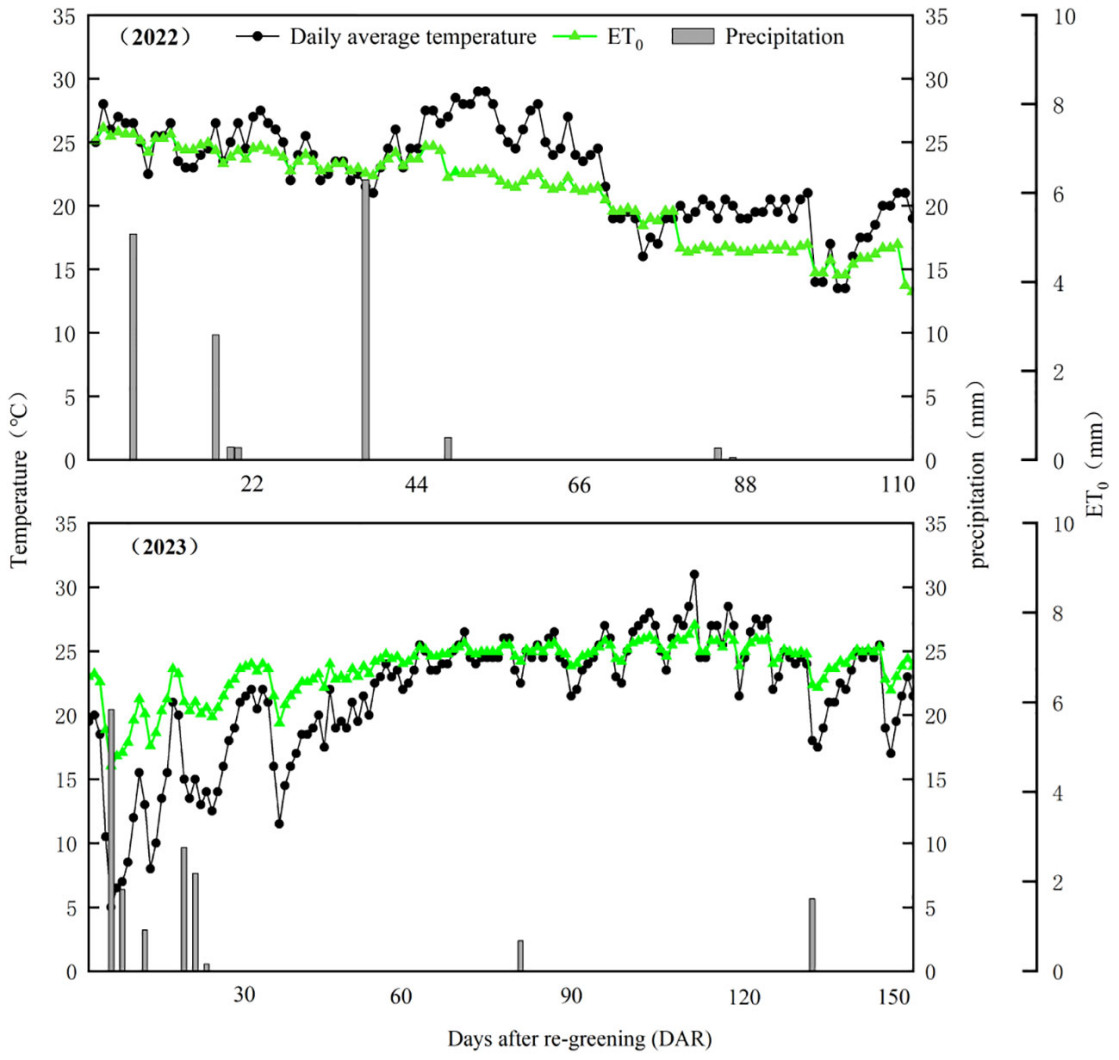
Precipitation, daily average temperature, and reference evapotranspiration (ET_0_) during the growing seasons of alfalfa in 2022 and 2023 in the experimental site. ET_0_ is calculated according to the methods of Allen et al. (1998) and Yan et al. (2021).

Field experiments were conducted in 2022 and 2023 in Botanical Garden No. 2 Village, Liangtian Town, Yinchuan City, Ningxia, China (106°18’E, 38°40’N) (**Figure 1**). The study site has a temperate continental climate with low rainfall and high evaporation. The average annual sunshine hours was 3,032 hours, the average annual frost-free period was 185 days, the average annual temperature was 8.7°C, and the average annual precipitation was 200 mm. The average annual potential evapotranspiration reached 1,694 mm. The soil type was aeolian sandy soil (91.76% of sand, 7.04% of silt, and 1.20% of clay). The pH of the surface soil (0-20 cm) was 8.62, the organic matter content was 4.67 g kg^−1^, the total nitrogen content was 0.31 g kg^−1^, the available phosphorus content was 2.44 mg kg^−1^, and the available potassium content was 81.42 mg kg^−1^. Temperature and precipitation data for both growing seasons came from a local weather station (**Figure 2**).

### 2.2 Experimental design

Three irrigation methods, namely flood irrigation, sprinkler irrigation, and subsurface drip irrigation, and two irrigation rates for each irrigation method were designed in this experiment. There were a total of six treatments arranged in a randomized complete block design, including (1) conventional flood irrigation (FI-12, irrigation volume: 1200 mm), (2) reduced flood irrigation (FI-8, irrigation volume: 880 mm), (3) conventional sprinkler irrigation (SI-8, irrigation volume: 880 mm), (4) reduced sprinkler irrigation (SI-5, irrigation volume: 520 mm), (5) conventional subsurface drip irrigation (DI-5, irrigation volume: 520 mm), (6) over subsurface drip irrigation (DI-8, irrigation volume: 880 mm). To ensure the same seedling emergence rate between different irrigation methods, sprinkler irrigation was conducted to promote seedling emergence in all experimental plots. After emergence, the sprinkler irrigation was stopped in the plots of FI and DI treatments. In the whole growing season, in addition to the irrigation for seedling emergence, sprinkler irrigation and subsurface drip irrigation were conducted four times at the seedling stage, branching stage, budding stage, and flowering stage for each crop (two crops in the first year, and four crops in the second year). For the FI treatments, irrigation was conducted at the seedling stage and budding stage of each crop (two crops in the first year, and four crops in the second year). In the first year, alfalfa plants were harvested two times, and the irrigation volume for the first and second crop accounted for 60% and 40% of the total irrigation volume of the year, respectively. In the second year, alfalfa plants were harvested four times, and the irrigation volume for each crop accounted for 25% (**Supplementary Table S1**).

**Figure 3:**
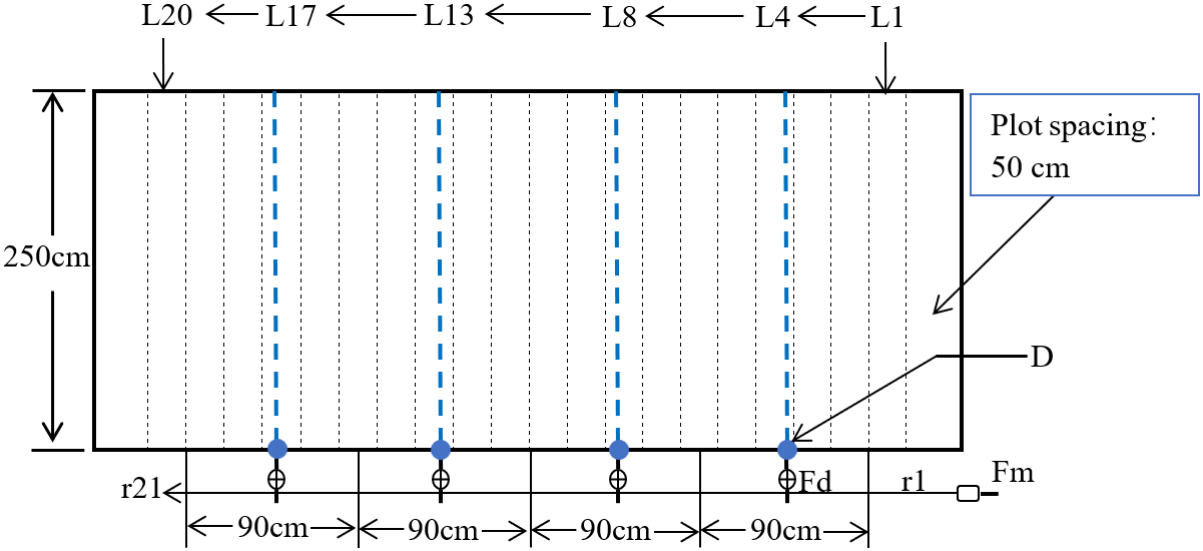
The layout of the pipes of subsurface drip irrigation in each plot. D is the location of the subsurface drip irrigation pipes; r1 - r21 are the rows of alfalfa; L1 - L20 are the spaces between alfalfa rows; Fd, fertilizer tank; Fm, flow meter.

The design of the sprinkler irrigation hoses and the arrangement of the pores followed Wang et al. (2021). The sprinkler irrigation hoses were 30 m long, the flow rate was 6.0 m^3^ h^−1^, and the spraying angle was 80°. The subsurface drip irrigation system consisted of a pump, a filter, a fertilizer tank, and water pipes. The pipes were imported from Germany, made of waste tire rubber and plastic through special technology. The inner diameter of the pipes was 13 mm, the wall thickness was 1.5 mm, and there were many micropores on the outer surface of the pipes. The outflow rate was 60-100 mL/(m·min), and the pressure was maintained at 0.06 MPa. According to the previous research on the parameters of subsurface drip irrigation system in alfalfa cultivation (Zhang et al., 2014), the spacing of the pipes in this study was set to 90 cm and the depth was 20 cm. The area of each plot was 12.5 m^2^ (2.5 m × 5 m), and the plot spacing was 50 cm. There were 21 rows of alfalfa in each plot, and the row spacing was 20 cm. The spaces between the alfalfa rows were named L1-L20 from left to right. Sprinkler irrigation hoses were laid in L10 (i.e. the width of the spraying range was 1 m on each side), and the subsurface drip irrigation pipes were buried in L4, L8, L13, L 17, and L20 (**Figure 3**). When conducting sprinkler and flood irrigation, baffles were used to reduce the mutual influence between plots. The baffles were 60 cm high and had an arc-shaped groove below. Besides, the baffles also had wedge-shaped tips, making them easy to insert into the fields. The irrigation water infiltrated quickly due to the sandy soil texture, thus the baffles were removed five hours after each irrigation and re-arranged before the next irrigation. Plots were separated by plastic films buried vertically (depth: 60 cm) to prevent mutual influences. For the plots of the FI treatments, a certain length of mulch film was left to wrap soils to form a 30 cm high ridge.

In this experiment, Juneng 401, an alfalfa variety suitable for planting in the arid desert area of Ningxia, China was used. Alfalfa seeds were sown in the spring of 2022, with a sowing rate of 15 kg/ha^−1^ and a row spacing of 20 cm. The total nitrogen application rate for each year was 225 kg/ha^−1^, of which 90 kg/ha^−1^ was applied before sowing and 135 kg/ha^−1^ was topdressed. Besides, 150 kg/ha^−1^ of P_2_O_5_ and 120 kg/ha^−1^ of K_2_O were also applied before sowing. For the nitrogen topdressings for the SI and DI treatments, nitrogen fertilizer was first dissolved in water and then applied through the corresponding irrigation system, while nitrogen fertilizer was sprinkled in the FI treatments. The fertilization time was consistent with that of local fields. The specific irrigation rates and nitrogen application rates of different crops of each treatment are shown in **Table 1**.

**Table 1:** Irrigation and nitrogen fertilization regimes.

Treatment	2022			2023				
	1st crop	2ndcrop	Total	1st crop	2nd crop	3rd crop	4th crop	Total
Irrigation volume (520 mm)	312	208	520	130	130	130	130	520
Irrigation volume (880 mm)	528	352	880	220	220	220	220	880
Irrigation volume (1200 mm)	720	480	1200	300	300	300	300	1200
Nitrogen topdressing rate (kg ha^−1^)	94.5	40.5	135.00	54.00	40.50	40.50	0.00	135.00

### 2.3 Sampling and measurement

#### 2.3.1 Yield components and seed yield

During the full flowering stage, 20 plants were randomly selected in each plot for labeling, and the number of inflorescences per plant (N_inflorescence_) and florets per inflorescence (N_floret_) were recorded. At the end of pod-setting stage, 20 pod-bearing branches were randomly selected in each plot, and the number of pods on each inflorescence (N_pod_) was recorded, Pod setting rate (%) was calculated using **Equation 1**:


(1) $$\begin{array}{c} \text{Pod setting rate }\left(\%\right)\\ \;=\text{ average number of pods per inflorescence }\\ /\text{average number of florets per inflorescence }\times \;100\text{\% } \end{array}$$

At the maturity stage in 2022 and 2023, three sampling subplots (1 m^2^ for each) were randomly selected in the center of each plot, and the pod-bearing branches of each subplot were counted. Then, the data was converted to the number of pod-bearing branches per square meter (N_branch_) according to the row spacing and plant spacing. Twenty representative pod-bearing inflorescences were selected from the sampled branches, and the number of seeds in each pod (N_seed_) was recorded. One thousand seeds were randomly selected from each treatment and weighed on a 1/1000 balance (4 replicates per treatment), followed by the calculation of 1000-seed weight by averaging. Ten plants in each plot were manually harvested, packed into net bags separately, air-dried, threshed, and weighed. The seed yield of each plant was recorded, and then the data were converted to the seed yield per unit area.

#### 2.3.2 Soil water consumption and water use efficiency

At the time of harvest, soil samples of the 0-20, 20-40, 40-60, 60-80, and 80-100 cm layer were collected with a soil sampler, and each-layer soil sample was divided into two parts. One part was used to measure the soil bulk density and SMC by drying method. The other part was used to measure soil nitrate nitrogen content. Soil water extraction was calculated as the difference in SMC (0-100 cm) between harvests. According to the method of Allen et al. (1998), due to the flat terrain and deep groundwater in this experimental area, groundwater recharge, surface runoff, and deep seepage were ignored to calculate the evapotranspiration over the whole growing season (ET) was calculated using **Equation 2**:


(2) ET=P +I –ΔWS

where P (mm) is the rainfall, I (mm) is the irrigation volume, and ΔWS (mm) is the change in soil water content from the beginning to the end of each crop.

Then, the ratio of the water consumption in each crop to the total ET (Ratio) and the ratio of hay yield to evapotranspiration (WUE) were calculated.

#### 2.3.3 Root distribution and root-shoot ratio (R/S)

At the initial flowering stage of the last crop of alfalfa in the second year, three representative quadrats (1 m × 1 m) were randomly selected from each plot, and the shoots were weighed after mowing (stubble height: 5 cm), and weighed again after drying to obtain the shoot biomass. Hay yield was calculated on the basis of shoot biomass (Fan et al., 2016). At the harvest time in 2022 and 2023, three sampling points were randomly selected in each plot (the interval between the three sampling points was 20 cm) to collect the 0-100 cm soil layer using a soil auger (diameter: 8 cm). Then, the roots were separated from the soils (Chang et al., 2016). Each sample was placed in a net bag. After washing off the soil with tap water and removing organic debris and other impurities, the root samples were scanned by a scanner (GT-F5201; Epson, Tokyo, Japan), and the RLD was determined using WinRHIZO Pro Vision 2009c software (Regent Instruments Inc., Québec, QC, Canada) (Xu et al., 2016). Finally, the root and shoot samples were wrapped with kraft paper and dried to constant weight to obtain root and shoot biomass. The ratio of root biomass to shoot biomass was the R/S.

#### 2.3.4 Soil nitrate nitrogen content and plant nitrogen accumulation and utilization

Soil sample was extracted with 2 mol L^−1^ KCl (soil: KCl solution = 1: 5), and then soil NO_3_^–^N content was measured by colorimetry using a spectrophotometer (UV-2102 PCS, Shanghai Spectrometer Co., Ltd., Shanghai, China) (Wang et al., 2015). The NO_3_^–^N content in the 0 - 100 cm soil layer was calculated as the sum of the NO_3_^–^N content in each layer of soil (Zhang et al., 2013). The total nitrogen content in the plants was determined using the Kjeldahl method (Dordas and Sioulas, 2009). Plant nitrogen accumulation and utilization were calculated using **Equations 3**–**5** (Ruisi et al., 2016):


(3) $$\text{N accumulation }\left(\text{NT}\right)\;={\text{D}}_{\text{M}}\times {\text{N}}_{\text{c}}$$

(4) $$\text{NUE}=\text{ HY}/{\text{N}}_{\text{f}}$$

(5) $$\text{NHI}={\text{N}}_{\text{G}}/{\text{N}}_{\text{T}}$$

Where D_M_ is the shoot biomass of mature plants, Nc is the concentration of nitrogen in the plant or grain, NUE is nitrogen use efficiency, HY is hay yield, N_f_ is the nitrogen fertilizer application rate, NHI is the nitrogen harvest index, N_G_ is the nitrogen accumulation in seeds, and N_T_ is the nitrogen accumulation of the whole plant at the mature stage.

#### 2.3.5 Ammonia volatilization

Soil NH_3_ volatilization was determined by aeration method (Yang et al., 2020). Three devices for measuring NH_3_ volatilization were placed between alfalfa rows of each treatment. The device was made of a polyvinyl chloride tube with a height of 15 cm and an inner diameter of 15 cm. Two sponges with a thickness of 3 cm and a diameter of 15 cm were pre-immersed in phosphate glycerol solution and placed in the above devices. Ammonia volatilization was measured daily in the first week after fertilization, and then measurement was taken every 3 - 7 days depending on the amount of NH_3_ volatilized, until NH_3_ volatilization was not detected. Samples collected in sponges were immediately extracted with 300 mL of potassium chloride solution (1 moL L^-1^) in a 500 mL container. The solution was shaken for 1 h and the concentration of NH_4_^+^-N was measured using a continuous segmented flow analyzer (AA3 HR AutoAnalyzer, SEAL Analytical Inc., Mequon, USA). Ammonia volatilization (kg N ha^-1^ d^-1^) was calculated using **Equation 6**:


(6) $$\text{Ammonia flux }=\frac{\text{M}}{\text{Area}\times \text{D}}\times 10^{-2}$$

where M (mg) is the amount of ammonia collected by a glycerin phosphate-soaked sponge, Area (m^2^) is the cross-sectional area of the polyvinyl chloride tube, and D is the time interval of ammonia collection.

### 2.4 Data analysis

All data were analyzed using the SPSS 17.0 software (SPSS Inc., Chicago, IL, USA), and the least significant difference (LSD) was conducted to compare the means of different treatments (*p*< 0.05). The correlations between R/S and yield components and WUE as well as between NH_3_ volatilization and SMC and NO_3_^−^-N content were analyzed using OriginPro 2024 software (OriginLab Corp., Northampton, MA, USA). Fitting and plotting were completed using OriginPro 2024 software (OriginLab Corp., Northampton, MA, USA).

## 3 Results

### 3.1 Seed yield and yield components

**Figure 4:**
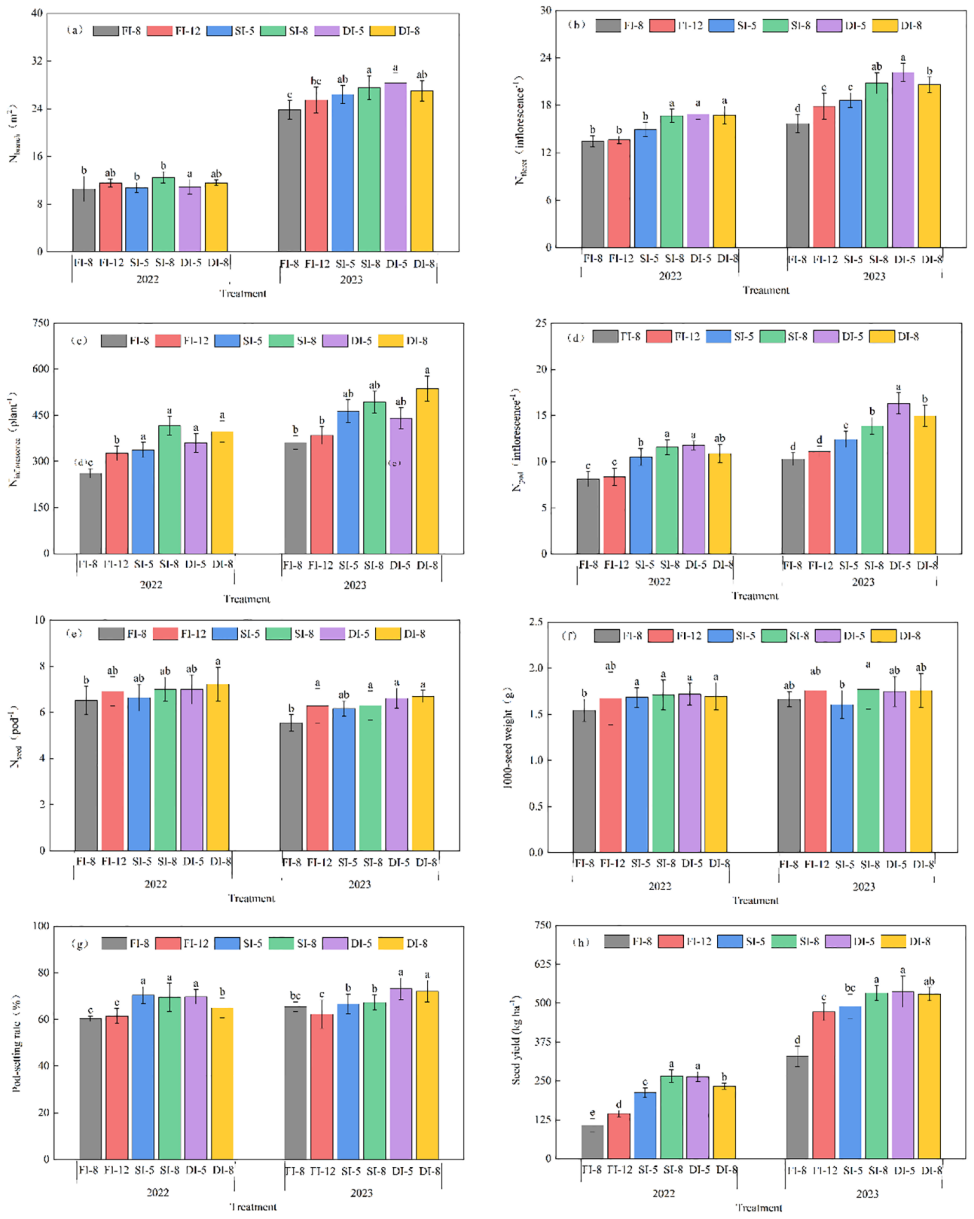
Impacts of different irrigation methods on the alfalfa yield components and seed yield in 2022 and 2023. **(a)**, N_branch_, the number of podbearing branches per square meter; **(b)**, N_floret_, florets per inflorescence; **(c)**, N_inflorescence_, the number of inflorescences per plant; **(d)**, N_pod_, the number of pods on each inflorescence; **(e)**, N_seed_, the number of seeds in each pod; **(f)**, 1000-seed weight; **(g)**, Pod-setting rate; **(h)**, Seed yield. FI-8, Reduced flood irrigation, 880 mm; FI-12, Conventional flood irrigation, 1200 mm; SI-5, Reduced sprinkler irrigation, 520 mm; SI-8, Conventional sprinkler irrigation, 880 mm; DI-5, Conventional subsurface drip irrigation, 880 mm; DI-8, Over subsurface drip irrigation, 880 mm. The same below. The error bar represents the standard deviation of the mean. Different lowercase letters indicate significant difference between treatments at p< 0.05.

The N_floret_, N_inflorescence_, N_pod_, and N_seed_ were significantly affected by the year (Y) and irrigation methods (M), and all indexes in 2023 were significantly higher than those in 2022 except for the N_seed_ (**Figures 4A–G**). The N_branch_, N_floret_, N_pod_, N_seed_, 1000-seed weight, and pod-setting rate were significantly affected by the Y and M interaction (Y×M).

The N_inflorescence_ and N_pod_ of the SI-5, SI-8, DI-5, and DI-8 treatments were significantly higher than those of the FI-8 and FI-12 treatments. Among them, the N_pod_ of the DI-5 treatment was the highest, increasing by 30.6% - 34.5% and 9.4% - 18.3% compared with that of the FI (FI-8 and FI-12) and SI (SI-5 and SI-8) treatments, respectively. The N_pod_ of the DI-5 treatment significantly increased by 7.9% compared with that of the DI-8 treatment.

Sprinkler irrigation and subsurface drip irrigation treatments increased alfalfa seed yield compared with traditional flood irrigation treatments (**Figure 4H**). When the volume of subsurface drip irrigation was 520 mm (DI-5), the seed yield reached the maximum, which was 1.57%-13.07% higher than that of DI-8 treatment.

### 3.2 Soil water consumption and water use efficiency

#### 3.2.1 Temporal and spatial variation of soil moisture content

**Figure 5:**
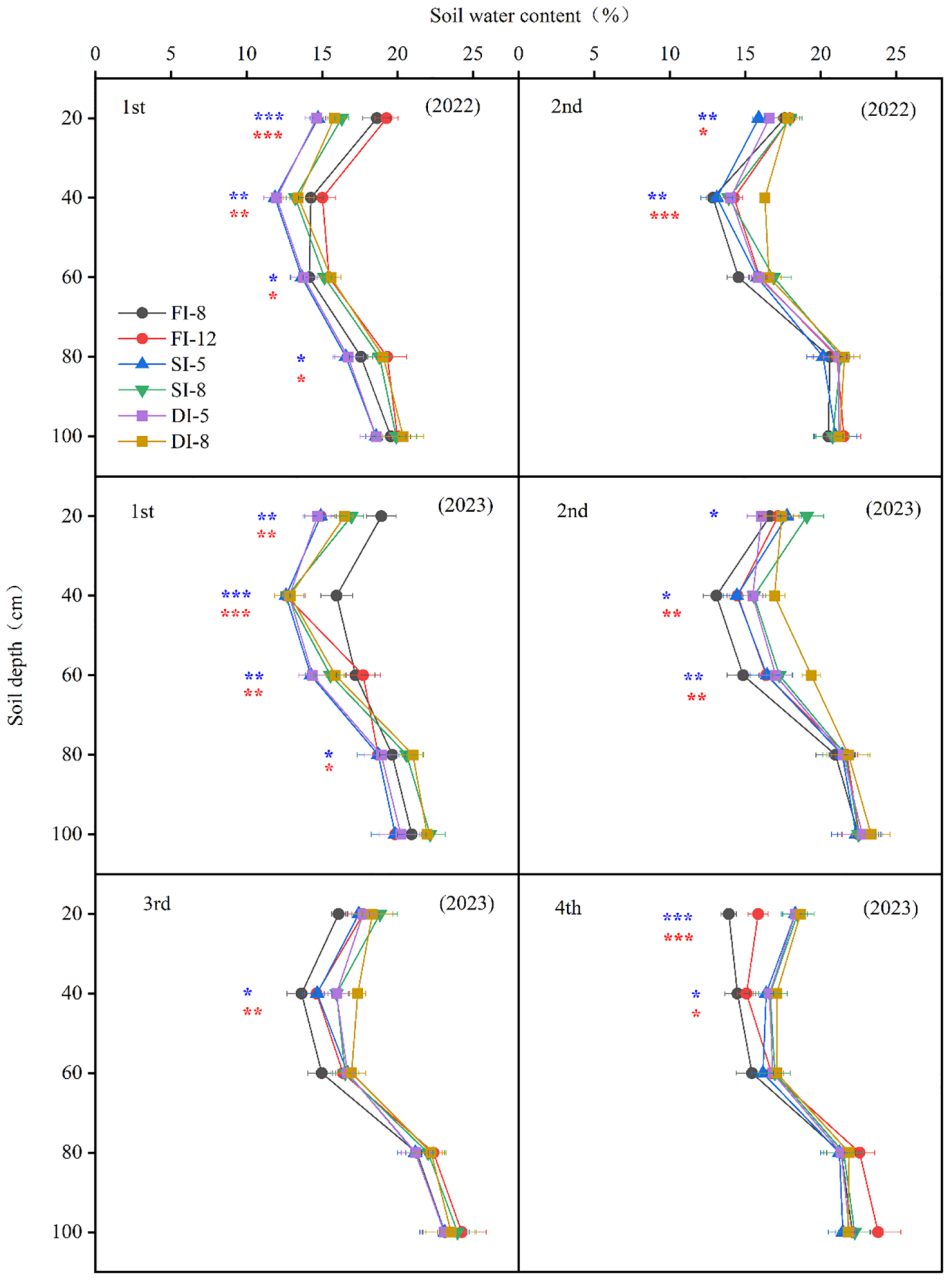
Impacts of different irrigation methods on the soil moisture content in 2022 and 2023. 1st: the first crop; 2nd: the second crop; 3rd: the third crop; 4th: the fourth crop. The red symbols *, **, and *** represent the significance of difference between subsurface drip irrigation (DI) and flood irrigation (FI) treatments at *p*< 0.05, *p*< 0.01, and *p<* 0.001, respectively, and the blue symbols *, **, and *** represent the significance of difference between sprinkler irrigation (SI) and FI treatments at *p*< 0.05, *p*< 0.01, and *p<* 0.001, respectively.

In the two years, the SMC of the same crop was similar, that is, the SMC decreased first and then increased with the increase of soil depth (**Figure 5**). The SMC of the 0-80 cm layer of the FI treatments (2022 and 2023) was significantly higher than that of SI and DI treatments. However, there was no significant difference in SMC in soil layers below 80 cm between treatments. In the second crop, the SMC in the upper soil layer (0-40 cm in 2022 and 0-60 cm in 2023) of the SI and DI treatments was significantly higher than that of the FI treatments, while there was no significant difference in SMC in the deeper soil layers (below 40 cm and below 60 cm, respectively) between treatments. In the third and fourth crops in 2023, the SMC in the upper soil layers (0-40 cm) of the SI and DI treatments was higher than that of the FI treatments, while there was no difference in the SMC in the deep soil layer (80-100 cm).

#### 3.2.2 Seasonal evapotranspiration and water use efficiency

Year (Y) significantly affected dry matter yield, year (Y) and irrigation method (M) significantly affected dry matter yield, ET, water consumption, and WUE, and Y×M significantly affected ET and WUE (**Table 2**). The dry matter yield of the DI treatments was significantly higher than that of the FI treatments, but there was no significant difference between DI treatments and SI-8 treatment. The ET of the SI-5 and DI-5 treatments significantly reduced compared with that of other treatments, and the ET of the FI-12 treatment was the highest. The water consumption of the SI-5 and DI-5 treatments in the first and second crops in 2022 and 2023 significantly reduced compared with that of the FI treatments, but the Ratio of the SI-5 treatment was significantly (44.88%-47.32%) higher than that of the DI-5 treatment. The WUE of the DI-5 treatment was second to that of the SI-5 treatment. The WUE of the DI-5 treatment increased by 61.9% and 43.8% in 2022 and 61.2% and 42.5% in 2023, compared with that of the FI-12 and SI-8 treatments, respectively.

**Table 2:** Effects of different irrigation methods on the total evapotranspiration (ET), water consumption in the first (1st) and second (2nd) crop, ratio of the water consumption in each crop to the ET (Ratio), and water use efficiency (WUE) in 2022 and 2023.

Year	Treatment	Dry matter yield (kg ha^-1^)	T (mm)	Water consumption (mm)				WUE (kg ha^−1^mm^−1^)
				1st crop	Ratio (%)	2nd crop	Ratio (%)	
2022	FI-8	6544.00c	947.27b	262.05b	27.66b	205.15b	21.66b	6.91e
	FI-12	7296.13bc	1238.20a	341.35a	27.57b	286.94a	23.17b	5.92f
	SI-5	7624.98b	297.53c	171.27c	57.56a	120.22c	40.41a	25.50b
	SI-8	7968.23ab	919.62b	262.77b	28.57b	207.51b	22.56b	8.73d
	DI-5	8693.31a	561.25c	174.09c	31.02b	117.38c	20.91b	15.53a
	DI-8	8875.16a	909.31b	260.45b	28.64b	205.50b	22.60b	9.76c
2023	FI-8	7856.69c	921.04b	256.86b	27.89ab	209.56b	22.75a	8.52e
	FI-12	9331.38b	1253.69a	330.92a	26.40b	284.53a	22.70ab	7.44f
	SI-5	9159.78b	559.84c	167.53c	29.92a	115.04c	20.55bc	16.37b
	SI-8	10167.02ab	920.27b	260.15b	28.27ab	205.69b	22.35abc	11.04d
	DI-5	10853.70a	565.84c	166.18c	29.37a	115.79c	20.46c	19.19a
	DI-8	10892.99a	917.52b	255.78b	27.88ab	205.20b	22.36abc	11.86c
Mean	FI-8	7200.35c	934.15b	259.45b	27.78bc	207.35b	22.20b	7.71d
	FI-12	8313.75b	1245.94a	336.14a	26.98c	285.74a	22.93b	6.68d
	SI-5	8392.38b	428.68d	169.40c	43.74a	117.63c	30.48a	20.94a
	SI-8	9067.62ab	919.95b	261.46b	28.42bc	206.60b	22.46b	9.88c
	DI-5	9773.50a	563.55c	170.14c	30.19b	116.58c	20.69b	17.36b
	DI-8	9884.08a	913.41b	258.11b	28.26bc	205.35b	22.48b	10.81c
ANOVA	Y	***	NS	NS	NS	NS	NS	NS
	M	**	***	***	***	***	***	***
	Y×M	NS	**	NS	NS	NS	NS	***

Ratio, the ratio of the water consumption in each crop to the total ET. Different lowercase letters indicate significant difference between treatments at *p*< 0.05.

### 3.3 Root distribution and root-shoot ratio

In the two growing seasons, the RLD of each treatment decreased with the increase of soil depth. The roots of the three irrigation treatments were mainly distributed in the 0-80 cm soil layer, and the RLD in the 0-60 cm soil layer of the FI and SI treatments was significantly higher than that of the DI treatments, while the RLD in 60-100 cm soil layer of the DI treatments was significantly higher than that of the FI and SI treatments. In 2022 and 2023, 95.8%-97.3%, 95.2%-96.6%, and 89.2%-90.1% of alfalfa root system of the FI, SI, and DI treatments were distributed in the 0-80 cm soil layer, respectively. In addition, the RLD in 2023, especially in the 0-40 cm soil layer, was significantly higher than that in 2022.

**Figure 6:**
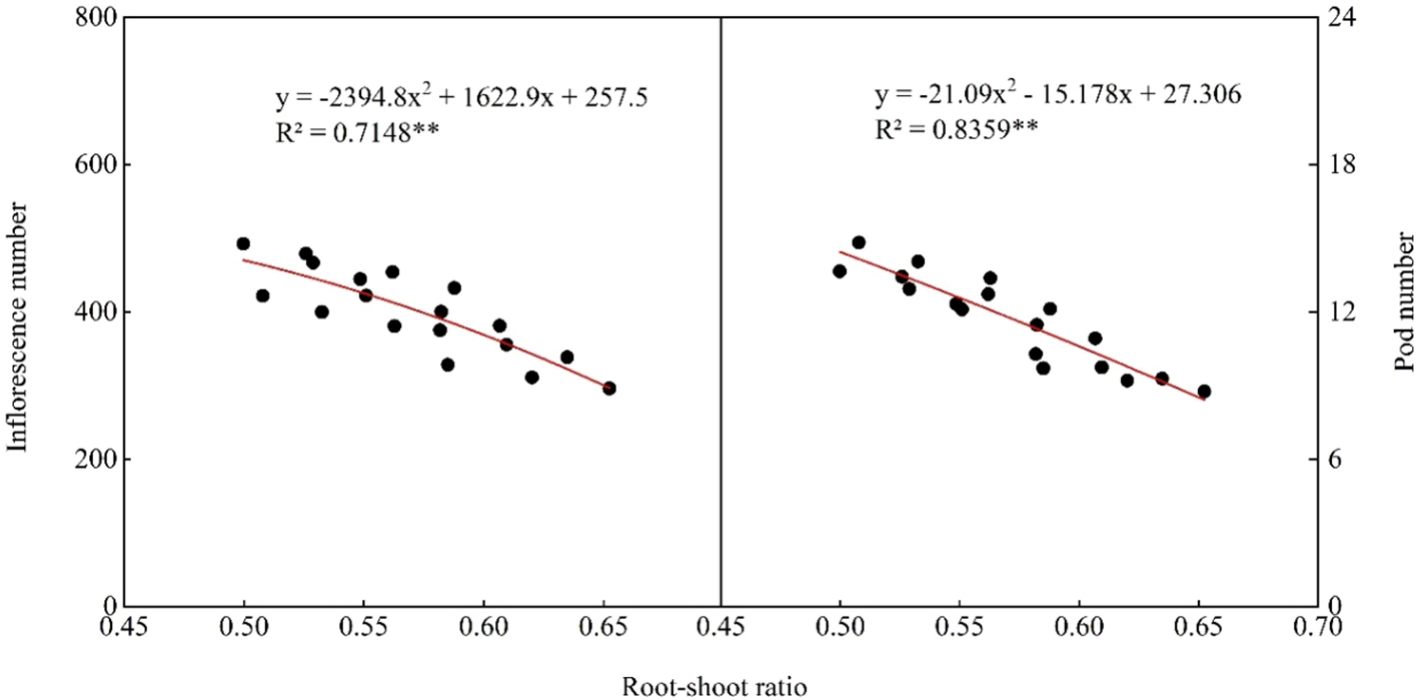
Correlation between root/shoot ratio (R/S) and number of inflorescences per plant and number of pods per inflorescence in 2022 and 2023. **p< 0.01.

**Figure 7:**
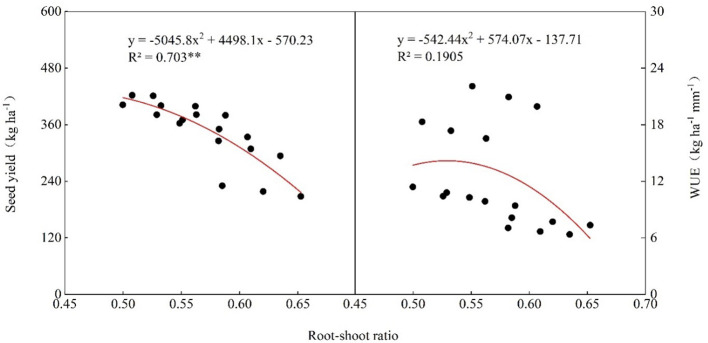
Correlation between root/shoot ratio (R/S), seed yield, and water use efficiency in 2022 and 2023. **p< 0.01.

The R/S of the DI treatments in the first crop (2022 and 2023) was significantly greater than that of the FI treatments. There was no significant difference in the R/S between FI, SI, and DI treatments in the second (2022 and 2023) and third (2023) crop. The R/S of the DI treatments in the fourth crop (2023) was significantly smaller than that of the FI and SI treatments. Under the same irrigation method, the R/S of the fourth crop (2023) of the FI-12 and SI-8 treatments was significantly lower than that of the FI-8 and SI-5 treatments. The R/S was negatively correlated with the N_inflorescence_, N_pod_, seed yield, and WUE (**Figures 6**, **7**).

### 3.4 Soil nitrogen residue and nitrogen utilization

#### 3.4.1 Temporal and spatial variation of soil nitrate nitrogen content

**Figure 8:**
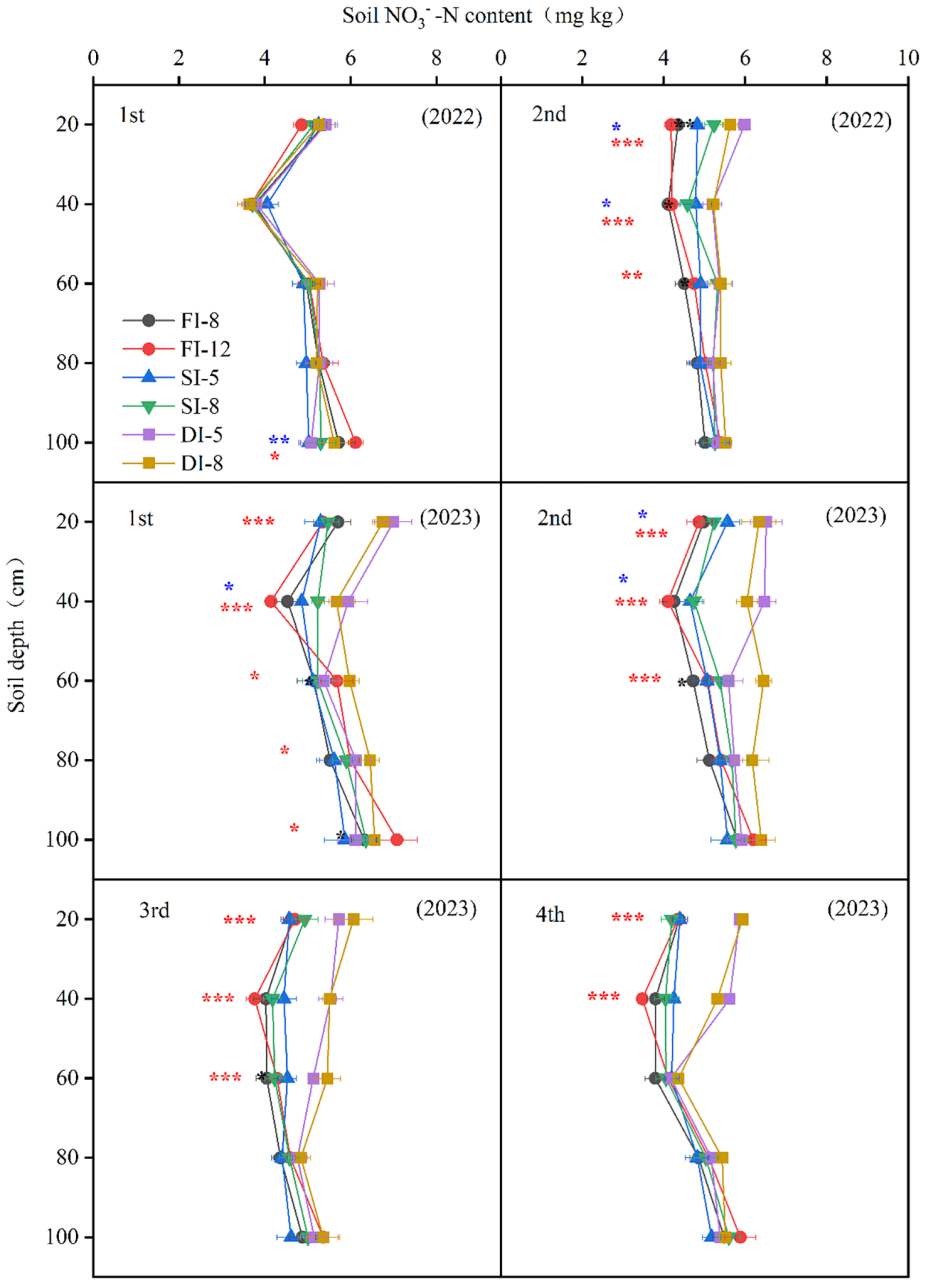
Effects of different irrigation methods on the soil nitrate nitrogen content in 2022 and 2023. 1st: first crop; 2nd: second crop; 3rd: third crop; 4th: fourth crop. The red symbols *, **, and *** represent the significance of difference between subsurface drip irrigation (DI) and flood irrigation (FI) treatments at *p*< 0.05, *p*< 0.01, and *p<* 0.001, respectively, and the blue symbols *, **, and *** represent the significance of difference between sprinkler irrigation (SI) and FI treatments at *p*< 0.05, *p*< 0.01, and *p<* 0.001, respectively.

The soil NO_3_^−^-N content of all treatments decreased first and then increased with the increase of soil depth (**Figure 8**). In the first crop in 2022, there was no significant difference in soil NO_3_^−^-N content in the 0-80 cm soil layer between treatments, the NO_3_^−^-N content in the 80-100 soil layer of the FI treatments was higher than that of the SI and DI treatments. In 2023, the NO_3_^−^-N content of the 0-40 cm soil layer of the DI treatments was significantly higher than that of the FI and SI treatments, and the NO_3_^−^-N content in the 80-100 soil layer of the DI treatments was significantly lower than that of the FI treatments. In the second crop (2022 and 2023), the NO_3_^−^-N content in the 0-60 cm soil layer of the DI treatments was significantly higher than that of the FI and SI treatments, while the NO_3_^−^-N content in soil layers below 60 cm was not significantly different among the treatments. In the third and fourth crops in 2023, the NO_3_^−^-N content in the 0-40 cm layer of the FI and SI treatments continued to decrease, while that of the DI treatments remained relatively higher than that of the FI and SI treatments. Besides, there was no difference in the NO_3_^−^-N content in the 80-100 cm layer between the third crop (2023) and the fourth crop (2023).

#### 3.4.2 Plant nitrogen accumulation and utilization

Year (Y) had a significant effect on the total nitrogen accumulation (TNA), NUE, and NHI (**Table 3**), and N and Y×M had a significant effect on the TNA, soil NO_3_^−^-N accumulation (SNA), NUE, and NHI. The TNA of the DI treatments significantly increased significantly compared with that of the FI treatments. There was no significant difference in SNA between treatments. The NUE of the DI-5 treatment significantly increased by 15.3% in 2022 and 13.2% in 2023 compared with that of the FI-12 treatment. In 2022, the NHI of the DI treatments was significantly lower than that of the SI treatments. However, in 2023, there was no significant difference in NHI between DI and SI treatments.

**Table 3:** Effects of different irrigation methods on the total nitrogen accumulation (TNA, kg ha^−1^), soil NO_3_^−^-N accumulation (SNA, kg ha^−1^), nitrogen use efficiency (NUE, kg kg^−1^), and nitrogen harvest index (NHI) in 2022 and 2023.

Year	Treatment	TNA	SNA	NUE	NHI
2022	FI-8	210.75d	346.29a	28.81d	0.15ab
	FI-12	262.71c	351.58a	32.45c	0.15a
	SI-5	282.95bc	349.71a	33.55c	0.16a
	SI-8	291.78bc	362.71a	35.00bc	0.15a
	DI-5	313.29ab	376.76a	38.31ab	0.13c
	DI-8	336.66a	373.66a	40.54a	0.14bc
2023	FI-8	315.62d	348.37a	34.59d	0.16a
	FI-12	366.69b	360.14a	41.50c	0.15ab
	SI-5	348.49bc	352.01a	40.30c	0.15ab
	SI-8	390.04ab	365.39a	44.66bc	0.15ab
	DI-5	419.40a	411.00a	47.84ab	0.14b
	DI-8	432.69a	418.01a	49.76a	0.14ab
Mean	FI-8	263.19d	347.33b	31.70d	0.15ab
	FI-12	314.70c	355.86b	36.98c	0.15ab
	SI-5	315.72c	350.86b	36.92c	0.16a
	SI-8	340.91bc	364.05ab	39.83bc	0.15ab
	DI-5	366.34ab	393.88a	43.08ab	0.14b
	DI-8	384.67a	395.84a	45.15a	0.14b
ANOVA	Y	***	NS	***	NS
	M	*	**	**	**
	Y×M	***	***	***	***

Different lowercase letters indicate significant difference between treatments at *p*< 0.05. *p< 0.05; **p< 0.01; ***p< 0.001, NS, *p* > 0.05.

### 3.5 Ammonia volatilization

**Figure 9:**
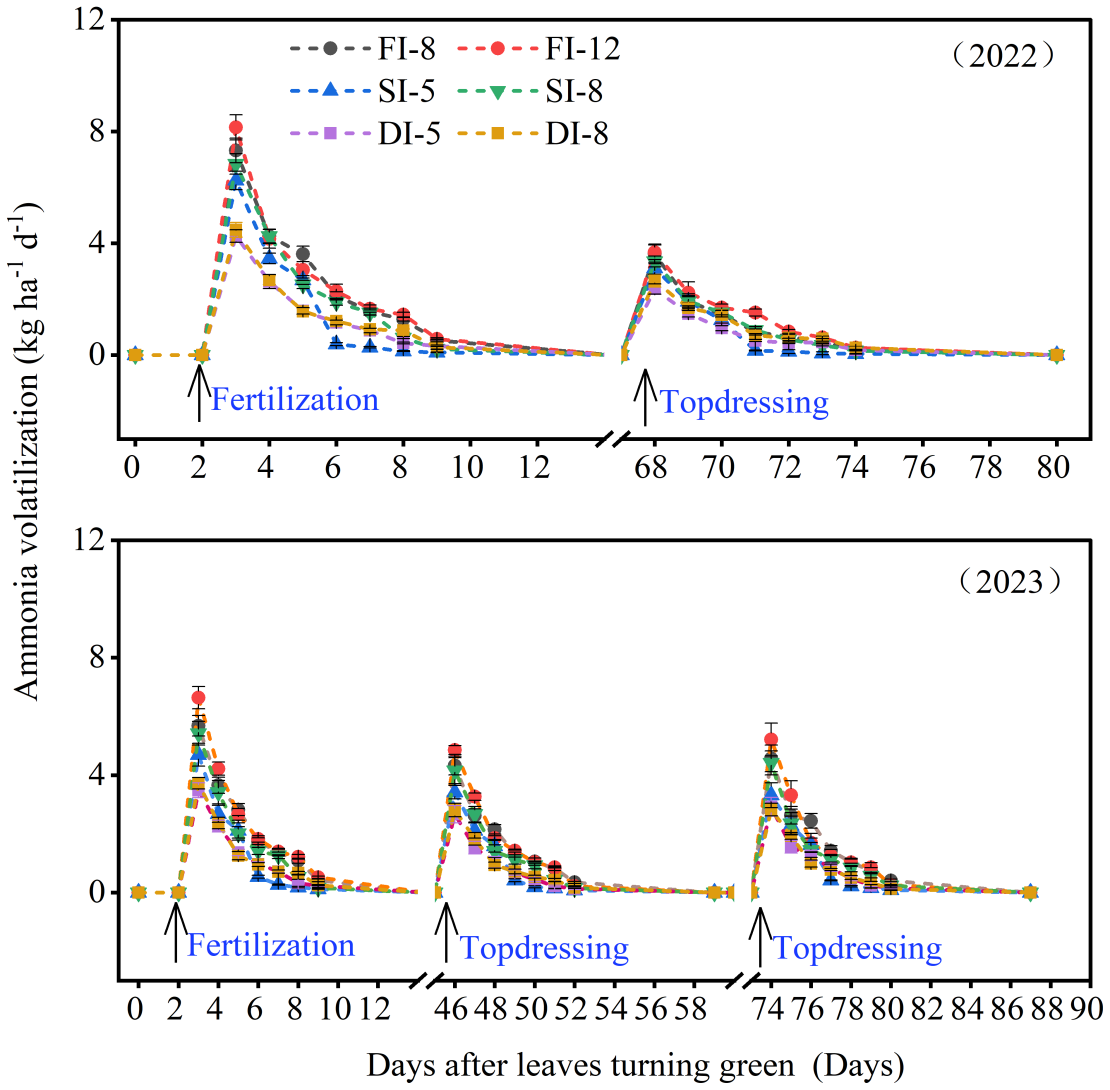
Effects of different irrigation methods on NH_3_ volatilization during alfalfa growing season in 2022 and 2023.

In 2022 and 2023, the NH_3_ volatilization of all treatments increased with the increase of nitrogen application rate (**Figure 9**). Ammonia volatilization showed an obvious peak after fertilization, and then decreased to a low level within 7 days. The highest NH_3_ volatilization of the three irrigation treatments appeared on the first day after fertilization. For the NH_3_ volatilization in the whole growing season (2022 and 2023), the NH_3_ volatilization of the DI treatments significantly reduced compared with that of the FI treatments. The NH_3_ volatilization of the DI-5 treatment decreased by 45.1% and 33.6% in 2022 and 48.5% and 35% in 2023 compared with that of the FI-12 and SI-8 treatments, respectively. There was no significant difference in NH_3_ volatilization between DI-5 and DI-8 treatments.

## 4 Discussion

Alfalfa seed yield is mainly determined by the N_branch_, the number of inflorescences per plant, N_floret_, N_seed_, and 1000-seed weight. A large number of studies have been conducted on the correlation between seed yield and its components. For example, Wang et al. (2006) showed that inflorescence number contributed the most to seed yield, followed by pod number. However, these yield components are susceptible to external environment and irrigation regimes (Wang et al., 2013). This study results showed that the inflorescence number per plant and N_pod_ were significantly affected by interannual variations and irrigation methods (**Table 2**). The number of inflorescences per plant and N_pod_ in 2023 was significantly higher than that in 2022, and the number of inflorescences and pods of the SI and DI treatments were higher than those of the FI treatments. Therefore, the seed yield of the SI and DI treatments was 29.7% - 32.6% higher than that of the FI treatments, and the seed yield of the DI-5 treatment with the minimum irrigation volume was the highest.

Many studies have shown that the WUE of crops can be improved by reducing ET or increasing yields (Igbadun et al., 2007; Gao et al., 2017). This study found that the ET of the SI-5 and DI-5 treatments significantly reduced and the alfalfa dry matter yield significantly increased, compared with those of other treatments (**Table 4**). Therefore, the WUE of the first two crops in 2022 and 2023 of the SI-5 and DI-5 treatments significantly increased by 68.1% and 61.5%, respectively, compared with that of the FI-12 treatment. In addition, during the first crop of two years, flood irrigation (FI-8, FI-12) could quickly provide sufficient water for the sandy soil with a low moisture content. As a result, the SMC in the 0-80 cm layer of the FI treatments was significantly higher than that of the SI and DI treatments. However, during the second crop in 2022 and the second, third, fourth crop in 2023, the SMC in the 0-40 cm layer of the SI and DI treatments was significantly higher than that of the FI treatments (**Figure 5**). This is due to the fact that sprinkler irrigation and subsurface drip irrigation use an irrigation strategy of small irrigation volume × multiple irrigation times, which can reduce the evaporation of soil surface water and facilitate water storage in the soil (Valentín et al., 2020). This indicates that sprinkler irrigation and subsurface drip irrigation could maintain a high moisture content of the root zone, and promote the utilization of deep soil water by roots, which is conducive to improving WUE, especially the subsurface drip irrigation. Therefore, in the soil environment with limited nutrients (i.e., sandy soil), subsurface drip irrigation inhibits root over growth by transferring the photosynthetic products originally allocated to the root system to the shoot. Therefore, subsurface drip irrigation is an efficient water-saving irrigation method.

**Table 4:** Effect of different irrigation methods on root/shoot ratio of alfalfa in 2022 and 2023.

Treatment	2022		2023			
	1st crop	2nd crop	1st crop	2nd crop	3rd crop	4th crop
FI-8	0.36b	0.48bc	0.51bc	0.58b	0.62bc	0.71a
FI-12	0.36b	0.51bc	0.53abc	0.56b	0.60b	0.65bc
SI-5	0.41ab	0.45c	0.47c	0.58b	0.63bc	0.68ab
SI-8	0.44a	0.50bc	0.51bc	0.59b	0.62bc	0.62c
DI-5	0.45a	0.56a	0.58a	0.65a	0.66ab	0.54d
DI-8	0.45a	0.54ab	0.56ab	0.61ab	0.69a	0.55d

Different lowercase letters indicate significant difference between treatments at *p*< 0.05.

**Figure 10:**
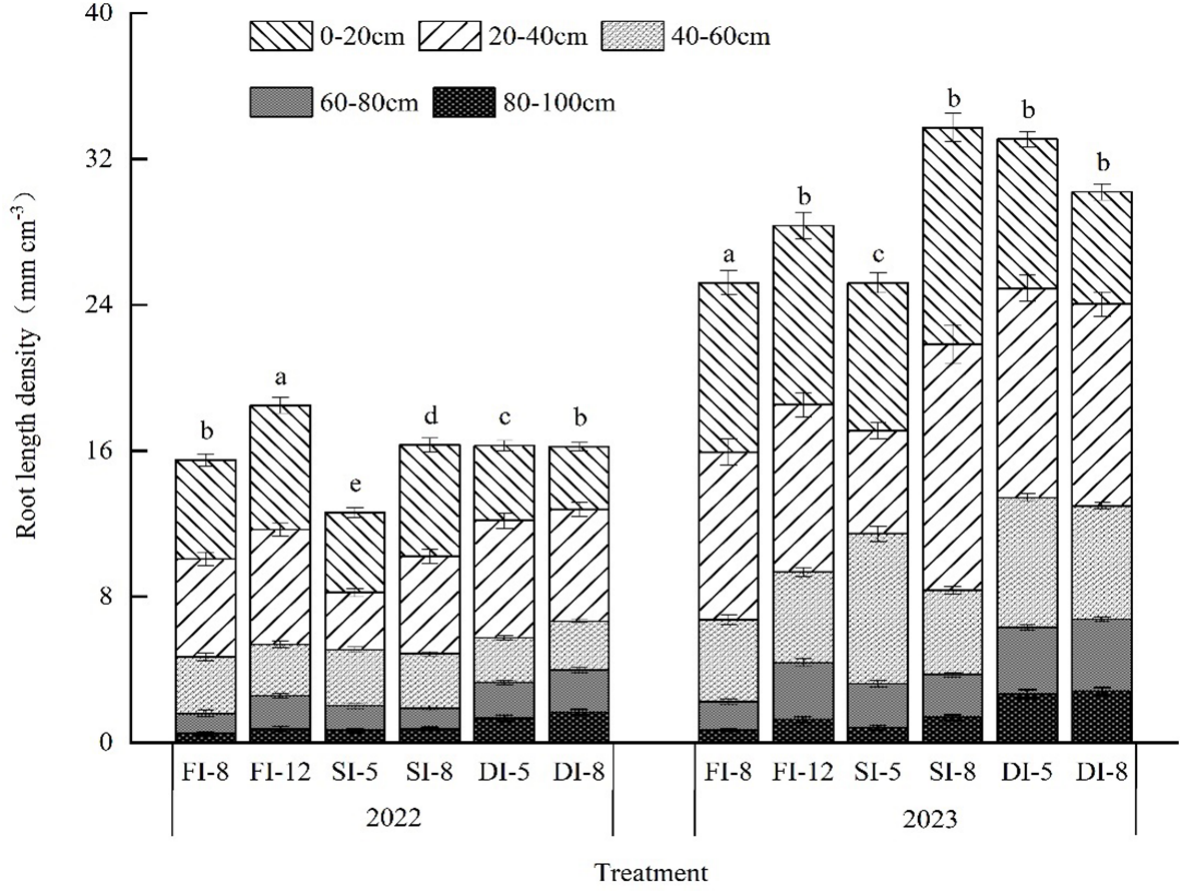
Root length density of the 0-100 cm soil layer at the maturity stage of alfalfa under different irrigation methods in 2022 and 2023. The error bar represents the standard deviation of the mean. Same letters indicate no significant difference.

Crop roots use soil moisture for growth, and soil moisture distribution has a great impact on root growth and distribution (Benjamin and Nielsen, 2006). In this study, alfalfa roots of all treatments were mainly distributed in the 0-80 cm soil layer. The RLD in the 0-60 cm soil layer of the FI and SI treatments was significantly higher than that of the DI treatments, but the RLD in the 60-100 cm soil layer of the DI treatments was significantly higher than that of the FI and SI treatments (**Figure 10**). This may be due to the fact that in the aeolian sandy soil environment in arid areas, the surface soil contains abundant water after flood irrigation and sprinkler irrigation, which stimulates the compensatory physiological response of the root system, that is, a large number of roots grow in the surface soil (Xu et al., 2010; Steinemann et al., 2015). The water and nitrogen availability in the 0-60 cm soil layer of the FI and SI treatments was higher than that of the DI treatments. However, the subsurface drip irrigation system supplies water for the alfalfa root zone, promotes alfalfa root growth in the deep soil (60-80 cm layer), thus increasing water utilization. It should be noted that there was a large change in RLD over the two years. In 2023, the second year of planting, the soil moisture and nutrient contents increased compared with those of 2022, and the number of pod-bearing branches and N_pod_ in alfalfa also increased (**Table 2**), leading to a significantly higher RLD (**Figure 10**).

The root and shoot of plants are a unified whole (Teixeira et al., 2008), and the R/S reflects the coordination of root and shoot growth (Luo et al., 2019). This study results showed that the R/S was negatively correlated with the N_inflorescence_, N_pod_, and seed yield (**Figures 6**, **7**). This indicates that an increase of R/S could inhibit the growth and podding of the aboveground reproductive organs. However, in the late growth stage (fourth crop in 2023), due to the weakening of the ability of roots to absorb water and nutrients, the irrigation water loss of the DI treatments was higher than that of the FI and SI treatments, resulting in a decrease in the R/S (**Table 3**). In the late growth stage of alfalfa (the second crop in 2022 and the fourth crop in 2023), the R/S of the DI treatments was smallest. This reduces the distribution of assimilates to the root system, and inhibits its over growth, thus coordinating the vegetative growth and reproductive growth in the late growth stage, and increasing the N_inflorescence_, N_pod_, and seed yield. In addition, the delayed maturation of alfalfa under over subsurface drip irrigation (Karamanos et al., 2009) led to dysregulation of vegetative and reproductive growth, significantly reducing the N_pod_ and seed yield (**Table 2**). The relationship between shoot and root not only determines the yield of crops, but also greatly affects the WUE of crops. This study found that the WUE decreased significantly with the increase of R/S (**Figure 7**). The R/S of the fourth crop of the DI-5 treatment was the lowest (**Table 3**), while a largest WUE was obtained at this time.

Soil nitrate nitrogen provides nitrogen nutrients for crops (Hawkesford et al., 2011), and its content is affected by fertilization and irrigation (Millar et al., 2018). In this study, the NO_3_^−^-N content of the FI treatments was significantly highest than that of the SI and DI treatments in the two years (**Figure 8**). Subsurface drip irrigation could reduce the NO_3_^−^-N leaching to the deep soil and ensure the nitrogen supply in the upper soil, increasing the uptake and utilization of soil nitrogen. This finally increased the NUE by 19.9%-27.2% and 11.6%-13.3% compared with flood irrigation and sprinkler irrigation, respectively (**Table 3**). Besides, under subsurface drip irrigation, water and nitrogen fertilizer were mainly supplied to the root zone (**Figures 5**, **8**). This promotes the root growth to the deep soil layers and the uptake of deep soil water and nitrogen, reducing the accumulation of NO_3_^−^-N in deep soil (**Figure 8**).

Canopy microenvironment has an important impact on crop yields, and subsurface drip irrigation can improve the canopy microenvironment and increase crop yields (Bhattarai et al., 2004; Valentín et al., 2020). In this study, the dry matter yield of the DI treatments was significantly higher than that of the FI treatments (**Table 4**). In addition, the TNA and NUE of plants of the DI treatments were higher than those of the FI and SI treatments (**Table 3**). This may be due to the fact that the timely water supply and uniform spatial and temporal distribution of water and fertilizers achieved by subsurface drip irrigation can promote seed nitrogen accumulation, increase NHI, delay leaf senescence, and improve the dry matter production capacity of leaves at maturity stage.

The NH_3_ volatilization accounts for at least 25% of the total nitrogen application rate (Pan et al., 2016; Xu et al., 2018) and is an important cause of low NUE (Beusen et al., 2008). In particular, a large NH_3_ volatilization not only waste resources, but also has adverse effects on the environment, causing soil acidification, water eutrophication, and increased atmospheric aerosols (PM_2.5_) (Liu et al., 2017). In this study, NH_3_ volatilization significantly increased after nitrogen application, but the effect of irrigation volume on NH_3_ volatilization was not significant, which was further confirmed by correlation analysis results, i.e., there was no correlation between NH_3_ volatilization and SMC (**Supplementary Figure S1**). In addition, NH_3_ volatilization was negatively correlated with soil NO_3_^−^-N content (**Supplementary Figure S1**). This may be due to the fact that the soil NO_3_^−^-N can be converted from ammonia nitrogen by nitrification. The accumulation of NO_3_^−^-N in soil can reduce the concentration of ammonia, which may reduce the NH_3_ volatilization. However, soil NO_3_^−^-N may also be converted to nitrogen (N_2_) or nitrous oxide (N_2_O) through denitrification (Li et al., 2013). This study also found that the NH_3_ volatilization of the DI treatments was significantly lower than that of the FI treatments. This is mainly due to that the integration of irrigation and fertilization of the subsurface drip fertigation is conducive to the conversion of urea by hydrolysis and the migration of urea to the root zone of crops, which promotes the uptake and utilization of nitrogen, thereby reducing the loss of nitrogen to a certain extent (Kulvir et al., 2022; Ana et al., 2024).

## 5 Conclusion

1. Appropriate subsurface drip irrigation (520 mm) can increase alfalfa seed yield by increasing the number of inflorescences and pods per plant compared with flood irrigation (conventional and reduced flood irrigation) and sprinkler irrigation (conventional and reduced sprinkler irrigation). The soil moisture content and root length density in the 60-100 soil layers below 60 cm of subsurface drip irrigation (conventional and excessive subsurface drip irrigation) were also higher, which promoted the utilization of water in deeper soil by roots and improved water use efficiency.
2. In the late growth stage (the fourth crop in 2023), the irrigation water loss under subsurface drip irrigation increased, and the root-shoot ratio decreased compared with that of flood irrigation and sprinkler irrigation. The small root-shoot ratio inhibited the over growth of the root system and promoted the distribution of photosynthetic products to the reproductive organs, thereby significantly increasing the inflorescence number per plant and pod number per inflorescence. It should be noted that over subsurface drip irrigation led to over growth of alfalfa shoots in the late growth stage, which reduced the number of pods and made the seed yield lower than that of conventional subsurface drip irrigation. Compared with flood irrigation and sprinkler irrigation, subsurface drip irrigation achieved higher water use efficiency and smaller root-shoot ratio in the late growth stage.
3. Subsurface drip irrigation reduced the leaching of NO_3_^−^-N to deeper soil and NH_3_ volatilization, and increased nitrogen use efficiency by 19.9%-27.2% and 11.6%-13.3% compared with flood irrigation and sprinkler irrigation, respectively.

In summary, subsurface drip irrigation is an efficient and environmentally friendly irrigation method. It could directly supply water and nitrogen to alfalfa roots, which is conducive to improving the seed yield, water and nitrogen use efficiency of alfalfa in the arid region of Northwest China, and reducing the NO_3_^−^-N leaching into the deeper soil. Therefore, subsurface drip irrigation with an irrigation volume of 520 mm can be widely applied in alfalfa planting in northwest China.

## Data Availability

The original contributions presented in the study are included in the article/**Supplementary Material**, further inquiries can be directed to the corresponding author/s.
