# Supplementary material for: Regulation of alfalfa growth, water and nitrogen utilization and distribution in arid region of Northwest China by optimizing irrigation method
Source: Front Plant Sci. 2025 Mar 12;16:1517398. doi: 10.3389/fpls.2025.1517398 (PMC11936995; doi:10.3389/fpls.2025.1517398)
Supplement: Supplementary file 1 [file DataSheet1.docx]

**Supplementary material**

**Table S1.** Harvesting, irrigation, and nitrogen application schedule for the alfalfa growing season (2022-2023).

| **Year** | **Growth**  **period** | **Days after re-greening**  **(Days)** | **Date** | **Irrigation** | **Nitrogen application** |
| --- | --- | --- | --- | --- | --- |
|  |  |  |  |  |  |
| **2022** | 1st | 0-64 | 10 May -12 July | 60% | 70% |
|  | 2nd | 65-108 | 13 July - 25 August | 40% | 30% |
| **2023** | 1st | 0-39 | 17 April-25 May | 25% | 40% |
|  | 2nd | 40-70 | 26 May-25 June | 25% | 30% |
|  | 3rd | 71-105 | 26 June-30 July | 25% | 30% |
|  | 4th | 106-145 | 31 July-8 September | 25% | - |

***** The values refer to the percentage of the total amount for different irrigation and N amounts.

**
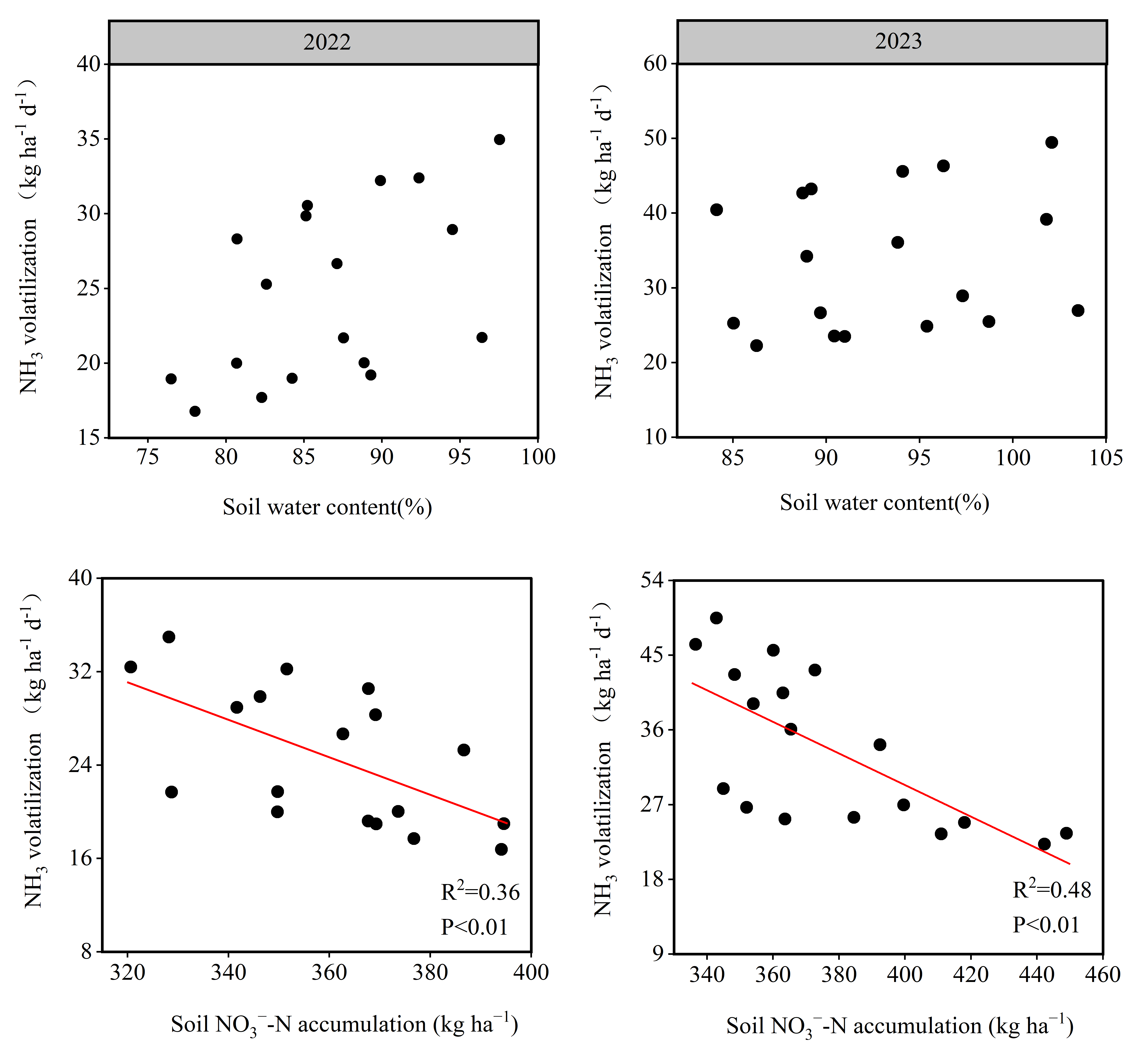
**

**Figure S1.** The relationship between NH_3_ volatilization and Soil water content， NO_3_^-^-N content.
